# Gaps in Study Design for Immune Parameter Research for Latent Tuberculosis Infection: A Systematic Review

**DOI:** 10.1155/2020/8074183

**Published:** 2020-04-21

**Authors:** Mariana Herrera, Cristian Vera, Yoav Keynan, Zulma Vanessa Rueda

**Affiliations:** ^1^Grupo de Epidemiología, Facultad Nacional de Salud Pública, Universidad de Antioquia, Medellín, Colombia; ^2^Grupo de Investigación en Salud Pública, Universidad Pontificia Bolivariana, Medellín, Colombia; ^3^Clínica Universitaria Bolivariana, Universidad Pontificia Bolivariana, Medellín, Colombia; ^4^Departments of Internal Medicine, Medical Microbiology & Infectious Diseases and Community Health Sciences, University of Manitoba, Winnipeg, Canada; ^5^Facultad de Medicina, Universidad Pontificia Bolivariana, Medellín, Colombia

## Abstract

**Background:**

Immune parameters (IP) have been extensively studied to distinguish between latent tuberculosis (LTBI) and active tuberculosis (TB).

**Objective:**

To determine the IP associated with LTBI, compared to active TB and individuals not infected by *M. tuberculosis* published in literature.

**Methods:**

We conducted a systematic search using Google Scholar and PubMed databases, combining the MeSH terms latent tuberculosis, *Mycobacterium tuberculosis*, cytokines, and biological markers, with the free terms, biomarkers and cytokines. Spanish, English, and Portuguese articles comparing the concentration of IP associated with LTBI, either in plasma/serum or in vitro, in adults and nonimmunocompromised versus individuals with TB or without *M. tuberculosis* infection between 2006 July and 2018 July were included. Two blinded reviewers carried out the searches, read the abstracts, and selected the articles for analysis. Participants' information, diagnostic criteria, IP, detection methods, and biases were collected.

**Results:**

We analyzed 36 articles (of 637 abstracts) with 93 different biomarkers in different samples. We found 24 parameters that were increased only in active TB (TGF-*α*, CSF3, CSF2, CCL1 [I-309], IL-7, TGF-*β*1, CCL3 [MIP-1*α*], sIL-2R, TNF-*β*, CCL7 [MCP-3], IFN-*α*, fractalkine, I-TAG, CCL8 [MCP-2], CCL21 [6Ckine], PDGF, IL-22, VEGF-A, LXA4, PGE2, PGF2*α*, sCD163, sCD14, and 15-Epi-LXA4), five were elevated in LTBI (IL-5, IL-17F, IL-1, CCL20 [MIP-3*α*], and ICAM-1), and two substances were increased among uninfected individuals (IL-23 and basic FGF). We found high heterogeneity between studies including failure to account for the time/illness of the individuals studied; varied samples and protocols; different clinical classification of TB; different laboratory methods for IP detection, which in turn leads to variable units of measurement and assay sensitivities; and selection bias regarding TST and booster effect. None of the studies adjusted the analysis for the effect of ethnicity.

**Conclusions:**

It is mandatory to harmonize the study of immune parameters for LTBI diagnosis. This systematic review is registered with PROSPERO CRD42017073289.

## 1. Background

Latent tuberculosis (LTBI) is defined as the presence of a positive tuberculin skin test (TST) or interferon-*γ* (IFN*γ*) release assays (IGRAs) in the absence of clinical or radiographic signs of disease. These accepted tests are imperfect for LTBI diagnosis for several reasons: (1) the sensitivity and specificity are between 71 and 82% for TST and 81 and 86% for IGRAs [1], (2) the sensitivity is reduced in immunocompromised patients, (3) there is inability to differentiate between LTBI and active tuberculosis (TB), (4) a positive TST or IGRA result does not automatically imply LTBI, as individuals who eliminate the infection successfully might still be TST- or IGRA-positive because of memory T cell responses, which partly explains the low predictive value of TST and IGRAs [1], and (5) genetic factors may impact test sensitivity as well as the susceptibility for acquisition of mycobacterial infection [2–4]. To date, there is no available diagnostic tool that allows diagnosis of LTBI and differentiates clearly between LTBI and active TB.

For the above-mentioned reasons, the World Health Organization, governments and nongovernmental organization, and private sector established as one of the priorities the identification of “what biomarkers or combinations of markers will help distinguish the various stages of the spectrum of LTBI (from sterilizing immunity to subclinical active disease)” [5].

The improvement in high-throughput cytokine measurement platforms has sparked enthusiasm for identification of novel pathways involved in the pathogenesis of TB that can inform development of assays for LTBI determination. In tuberculous infection, some important immune molecules are known to play a pivotal role in the protective response against the bacteria. Among the main ones described are IFN-*γ*, produced by T CD4+, CD8+, and NK cells, and IL-1 and TNF-*α*, secreted by macrophages and lymphocytes, known to prevent the growth and multiplication of mycobacteria in host cells [6, 7]. However, additional biomarkers such as IL-2, IL-5, IL-10, IL-1RA, and MCP have been studied for their ability to differentiate between the LTBI and active TB [8], and it is believed that the cellular and immune profile expressed during tuberculous infection depends to a great extent on the stage of disease, i.e., LTBI or active, where immune biomarkers present in blood could have the ability to differentiate with greater precision between both stages [9].

Despite advances in the study of immune parameters, there are pervasive limitations in the analysis and conclusions of many of these studies. Cytokine/chemokine expression is affected by ethnicity [2, 10], cell simulation protocols (or no stimulation) [11–13], time of LTBI (which in most cases is impossible to quantify), and if the comparison group is people with TB, the clinical manifestations of disease (pulmonary vs. extrapulmonary TB) [14].

In order to identify which immune parameters are increased exclusively in LTBI, in addition to finding gaps in knowledge and study design of previous published papers, we performed a systematic review. The question posed is the following: what are the cytokines associated with LTBI, compared to cytokines expressed among individuals with active TB and those not infected by *M. tuberculosis*?

## 2. Methods

According to the Preferred Reporting Items for Systematic reviews and Meta-Analysis protocols (PRISMA-P), this systematic review was registered with the International Prospective Register of Systematic Reviews (PROSPERO) on August 31, 2017 (registration number CRD42017073289).

### 2.1. Eligibility Criteria

Studies were selected according to the following criteria.

#### 2.1.1. Study Designs

We included clinical trials, prospective and retrospective comparative cohorts, and case-control and cross-sectional studies. We excluded descriptive studies, case reports and series, and reviews.

#### 2.1.2. Participants

The participants are those from articles published between January 2006 and July 2018, which compared people with LTBI with 18 years or older, or adults and children, without any immunocompromising medical conditions, versus individuals with active TB or without *M. tuberculosis* infection under the same conditions. We excluded manuscripts assessing the production of IFN-*γ* as part of the evaluation of IGRAs, which were performed in animal models, immunocompromised individuals, and studies exclusively conducted in children.

#### 2.1.3. Exposure

Articles that evaluated the expression of cytokines associated with LTBI, either in plasma or in vitro, with or without stimulation of mycobacterial antigens were included. The antigens used to perform cell stimulation were not restricted.

#### 2.1.4. Comparators

The comparators are the expression of cytokines associated with active TB confirmed by clinical and epidemiological contact, X-rays, and/or laboratory and/or subjects with no evidence of *M. tuberculosis* infection, evidenced by negative results of the tuberculin skin test or interferon-gamma release assays.

#### 2.1.5. Outcome

People with LTBI were compared to those with active TB or with no evidence of *M. tuberculosis*.

#### 2.1.6. Timing

There was no restriction on the length of follow-up for clinical trials or cohort studies.

#### 2.1.7. Setting

There was no restriction on the type of setting.

#### 2.1.8. Language

Articles in English, Spanish, or Portuguese were included.

### 2.2. Information Sources

Search for original articles utilized two electronic databases: Google Scholar and PubMed.

To identify additional literature, the reference list of all papers was reviewed, and we followed the same process for abstract reviewing and data extraction as we did for papers identified by electronic search. Articles suggested by the reviewers, not detected in the previous searches, were also included.

### 2.3. Search Strategy

Papers published between July 2006 and July 2018 were included. We used the following MeSH terms in English, Spanish, and Portuguese languages: latent tuberculosis, *Mycobacterium tuberculosis*, cytokines, and biological markers. In addition, we used the free terms biomarkers and cytokines. Additional file 1 contains the search strategies used.

### 2.4. Study Selection, Data Collection Process, and Data Items

Once the articles were identified using each of the search strategies, we proceeded with the elimination of duplicate items. Subsequently, the titles and abstracts of all manuscripts identified by two independent evaluators were reviewed according to the selection criteria. All disagreements between the two reviewers were resolved with a third evaluator by consensus. Articles that met the selection criteria were read completely by the same reviewers, blinded and independently.

The data extracted and typed in an Excel file from the selected articles were the following: consecutive number of the article (whole number assigned by investigators), article title, year, first author, journal, study country of origin, outcome or result reported in the article, type of study population (special feature), number of patients in the intervention or comparison group, follow-up in each group, type of control or unexposed population, number of patients in the control or unexposed group, follow-up in the control or nonexposed group (months), age, sex (female percentage), active TB diagnostic method, LTBI diagnostic method, LTBI time, immune parameters studied, increased IP (with and without statistical differences) (the group in which the IP was increased is reported first), IP that remained normal, decreased IP (with or without statistical differences), IP concentration values, the level of confidence they used in their statistical analyses (90%, 95%, and 99%), method of detection of IP, if ethnicity was reported, the study populations, type of study, quality of the study (see below), bias (types of bias), proportion of BCG vaccine, conflict of interest statement, and other important findings such as the cell stimulation used (times and antigens used).

We conducted a pilot study for the search strategies, abstract reviewing, and data extraction of full-text articles to standardize all process and concepts before starting each step. A third reviewer was in charge of comparing the files to identify disagreements at each step of the process. A fourth reviewer participated in the validation of the biological findings, only at the end of the full-data extraction for included papers to avoid investigator bias.

### 2.5. Risk of Bias in Individual Studies

Selection bias was controlled through the application of inclusion and exclusion criteria to eligible titles and/or summaries; likewise, possible information biases were controlled by the independent revision of two observers, where at the end of the review, a third reviewer compared their findings. The risk of bias of the studies was assessed using the Newcastle-Ottawa scales for case-control and cohort studies [15] and the National Institutes of Health (NIH) evaluation scale for observational studies [16]. The Jadad scale was applied to evaluation of clinical trials [17] (Additional files 3 and 4).

The Newcastle-Ottawa scale evaluates four main points: population, that is, the choice of cases or exposed people, and controls or not exposed; the measurement of the outcome and exposure; and the comparability between groups [15]. Similarly, the NIH scale is based on 14 questions that include the clear definition of the objective, the population (including the sample size), the measurement of dependent and independent variables, and the control of the confounders [16].

For both scales, one or two points are given when a study complies with the evaluated requirements (comparability for Newcastle-Ottawa). This final score determines the risk of bias: high risk (0-2 points), moderate (between 3 and 6 points), and low risk of bias (≥7 points).

### 2.6. Summary Measures

Due to the clinical heterogeneity of the population, the samples and the stimulation protocol used, the multiple techniques used for immune parameter detection, the different units reported for the substances, and the differences in the diagnosis of LTBI and active TB, it is was deemed inadequate to perform a meta-analysis [18, 19]. Therefore, we report the systematic review with a qualitative synthesis of the papers.

## 3. Results

### 3.1. Articles

**Figure 1:**
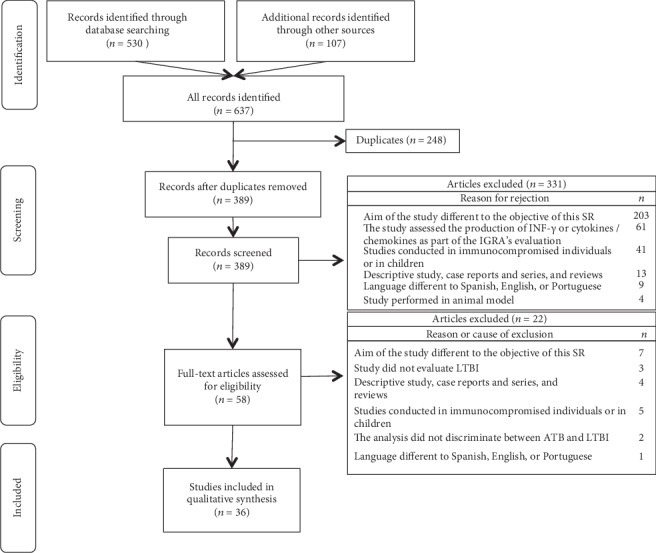
PRISMA diagram showing the results of systematic searches and articles analyzed. Legend: ATB: active tuberculosis; LTBI: latent tuberculosis infection.

Upon searching according to the keywords, 637 relevant articles were retrieved; among them, 58 met the selection criteria and were read in full text. At the end, 36 met all criteria and were included in the systematic review (Figure 1). The excluded articles and the reasons for exclusion are provided in Additional file 2.

Publications included 34 cross-sectional and 2 cohort studies, the latter with follow-up for 6 and 24 months after baseline sampling.

### 3.2. Participants

Most of the studies evaluated individuals with active TB treatment or within hospital programs. Their community or family contacts or voluntary hospital or community-based controls with or without TB infection served as controls. Five studies were conducted in healthcare workers, four in places endemic for TB, and one from a region with a high rate of malnutrition. The minimum and maximum numbers of subjects included in the studies were 7 and 148 in the LTBI group, 10 and 147 in the active TB group, and 8 and 168 in the noninfected group.  Table 1 describes the characteristics of the population included in each study.

### 3.3. BCG Vaccination Status

Among the 36 included articles, 22 reported BCG vaccination [20–42]. The proportion of BCG vaccination was similar among the groups with LTBI, active TB, and noninfected individuals. Seven papers [26, 28, 36, 38, 42–44] compared the statistical difference between those with and without vaccination, and only two reported statistical significance [45, 20], having a lower percentage in the active TB group compared to healthy controls (healthy persons with no known risk of TB exposure), TB-exposed persons with QFT-negative results, and people with LTBI. One article reported no significant associations between levels of cytokines and BCG scar [21], and one included BCG status in the multinomial regression model getting an adjusted odds ratio increased for TNF-*α* and IL-6 [22].

### 3.4. Evaluation of Conversion to LTBI and Progression to Active TB

The majority of the studies did not evaluate progression to active TB and conversion to LTBI. Most of literatures that we reviewed were cross-sectional studies that only have the prevalence or frequency of LTBI [22, 21] and active TB and those who are TST-negative or IGRA-negative. We found two cohort studies, and only one of them evaluated risk of LTBI conversion and progression to diseases, but it reported that they have a low number of convertor (2/101 individuals) and any progressor to active TB [21]; therefore, it is not feasible to identify cytokines that allow to identify progression to either.

### 3.5. Diagnostic Methods for LTBI and Active TB

The methods used for LTBI diagnosis were the following: 18 studies used TST and IGRAs, nine relied on TST alone, one used TST or IGRAs plus clinical criteria, and eight utilized IGRAs alone. In studies where the two tests were used, the discordant results between the two tests are evident.

In order to establish the diagnosis of active TB, researchers used one or a combination of the following criteria: history of contact with a TB case, smear (Ziehl-Neelsen or auramine rhodamine stain), culture, clinical diagnosis, molecular test, pathology, and/or X-rays.

### 3.6. Measurement of Immune Parameters

In total, 93 substances (Table 2) were studied, including growth factors; interferons; receptors; tumor necrosis factors; alpha, beta, and delta chemokines; interleukins; and others like sCD40L, MIF, and sCD14.

**Figure 2:**
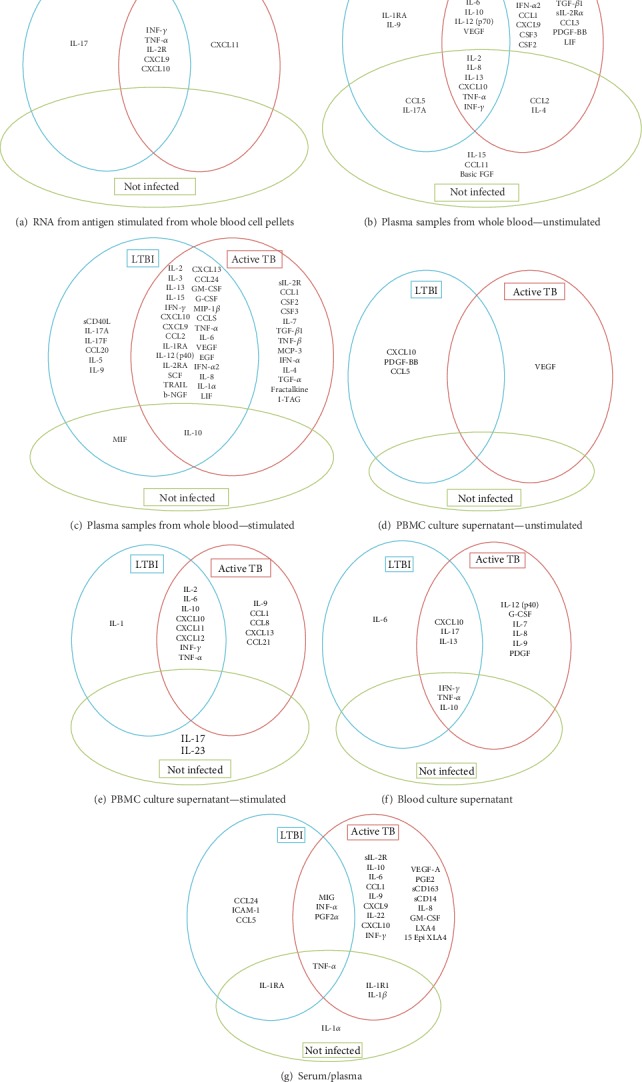
(a–g) Immune cytokine/chemokine mediators statistically different reported in active TB, latent tuberculosis infection, and noninfected individuals in each sample type.

Of these, 24 substances were increased only in active TB, five increased only in the LTBI group, and two increased in uninfected individuals, regardless of the sample analyzed (Figure 2).

Table 1 shows all the antigens and times used for *ex vivo* stimulation and cytokines whose concentration was statistically different, by each group. The most frequently measured mediators were IL-6, IL-10, IL-2, TNF-*α*, INF-*γ*, and IP-10.

Most of the studies reported mediators after *ex vivo* stimulation, with most using supernatants of interferon-gamma release assays.

Only eight papers evaluated serum or plasma samples without stimulation. There were no differences between them; only MIG was increased in plasma samples in TB patients, but elevated in serum samples in both, LTBI and active TB.

Among evaluated immune mediators, most were measured in plasma samples from stimulated or unstimulated (as controls) whole blood (Figures 2(a)–2(g)). Other samples utilized were supernatants from PBMC cultures and whole blood culture and RNA from blood cells.

The stimulation antigens used were ESAT-6, PPD, CFP-10, TB7.7 (Rv2654), TB10.4, PE35 (Rv3872), PPE68 (Rv3873), Rv262, FbpB, E6C10, Rv2031, protein fraction 11_24 (Rv2626c), H37Rv soluble antigens, DosR Rv1737c, Rv2029c, Rv2628, Rpf Rv0867c Rv2389c, and inactivated bacteria (Table 1).

The concentration of biomarkers involved in the immune response is dependent on the type of protocol used for *in vitro* stimulation and the sample evaluated and has high variability between studies. Among the five substances exclusively elevated in LTBI and the two elevated in uninfected individuals, there was inconsistency in the samples processed throughout the studies. For example, IL-5 was evaluated in plasma (1 article), plasma samples from stimulated whole blood (11 articles), PBMC culture supernatant (3 articles), and blood culture supernatant (1 article) but was only increased in two papers that used plasma samples from stimulated whole blood (2/11 articles). In the case of active TB, one substance (CCL1/I-309) was elevated in four different sample types, one (IL-7) in three sample types, and the rest in two (usually plasma samples from whole blood unstimulated and stimulated) or one sample type.

ELISA (*n* = 17) and microbead-based method (*n* = 20) were the most frequently used methods for IP measurement (Table 1). Some of the articles used both methods.  Table 1 describes details regarding laboratory measurements.

### 3.7. Risk of Bias of Included Articles

Of the 36 articles reviewed using the Newcastle-Ottawa scales and the Quality Assessment Scale for NIH observational studies, 7 articles had low risk of bias, 29 moderate, and none high risk of bias. Only one study out of the 36 performed calculation of the sample size and took into account the statistical power of their results (Additional files 3 and 4).

### 3.8. Biases

The main bias identified in the articles reviewed was the absence of a second administration of the tuberculin skin test to detect a possible booster effect, thus leading to the potential inclusion of individuals with false negative results of the TST. Another bias was the analysis of patients with pulmonary and extrapulmonary TB in the same group of active TB as the underlying immune competence and immune response may vary between localized or disseminated disease. In addition, children and adults were included in some studies; however, the analysis was not stratified for each population. Finally, patients with pulmonary TB were included in different phases of treatment; some studies included individuals that completed treatment at the time of IP measurement. The declining microbial burden during or at the end of therapy may contribute to false negative results (Additional files 3 and 4).

Only 4 of the reports took into account the study origin and population's ethnicity as a confounding factor, and these were evaluated by self-reported ethnicity [28, 38, 48, 52].

Of the included manuscripts, 27 articles provided a declaration of conflicts of interest (S3 and S4).

## 4. Discussion

The immune response against infection and disease caused by *M. tuberculosis* is mainly mediated by the recruitment and activation of T cells and macrophages, which in turn are regulated by multiple immune mediators such as interleukins and chemokines, possessing a diverse pro- and anti-inflammatory property. The success of the immune response in halting the acquisition of *M. tuberculosis* is influenced by a myriad of environmental, microbial, and host factors. The host response is measured in order to determine *M. tuberculosis* infection in the form of skin tests or IGRAs, but this approach is limited by the inability to differentiate LTBI from active TB infection. The ability to refine diagnostics by using assays that incorporate measurement of multiple biomarkers will be critical in order to stride towards TB control and eventual elimination.

Most of the studies analyzed in this review focused on the main pro- and anti-inflammatory interleukins involved in the immune response, mediated mainly by Th1 and Th2 lymphocytes; a few others expanded the markers measured to include chemokine-like substances, growth factors, and receptors as part of the search for new diagnostic biomarkers that can discriminate between LTBI and active TB.

Several immune mediators in addition to INF-*γ* have been identified. The most frequently evaluated markers are the cytokines IL-6, IL-10, IL-2, TNF-*α*, and IP-10. Although the response to TB is reliant on Th1 (TNF-*α*, INF-*γ*, and IL-2), this has been expanded by the addition of Th2 signature cytokine profile such as IL-6 and IL-10.

**Figure 3:**
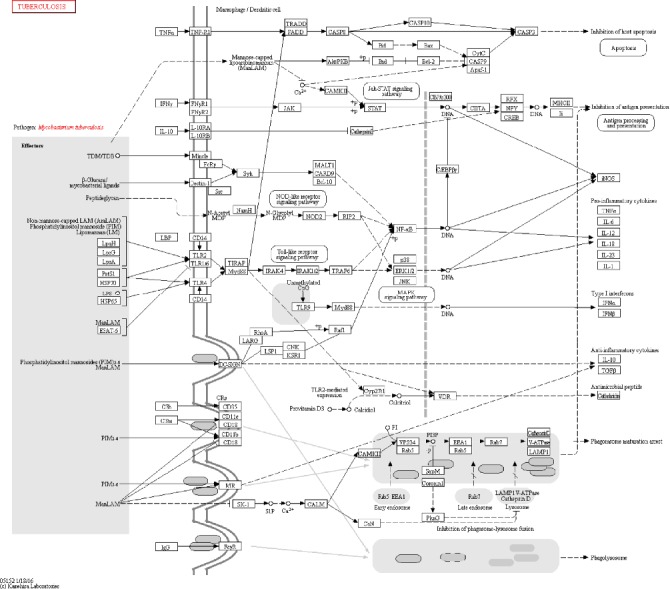
KEGG pathway, highlighting some of the pathways and mediators identified in the reviewed studies. The use of this figure was granted by copyright permission of KEGG [57], and the journal has a copy of the approval.

Elevated immune mediators and markers that were only detected in active TB share chemoattractant functions involved in trafficking of cells involved in the immune response, among which are T lymphocytes (CD4+ and CD8+), macrophages, dendritic cells, basophils, and eosinophils. These cytokines affect cell growth, maturation, and differentiation (Additional file 5). In LTBI, only interleukins IL17F and IL-5, associated with effector T cell profiles, are overexpressed. The effect of the cytokines found overexpressed in LTBI is related to the increased production of immune substances, chemoattraction, multiplication, and activation of lymphoid cells [56]. Of the cytokines identified in the systematic review, three (IL-12 and TGF-*β* for active TB and IL-23 for uninfected individuals) are well-established markers involved in immune response to mycobacterial infection (Figure 3: available at http://www.genome.jp/kegg/pathway.html) [57].

While most attention has been directed to immune cells, some of the immune substances that participate in the response to *M. tuberculosis* are produced by epithelial cells, which play a fundamental role in the initiation and expansion of host defense mechanisms in the lung, providing protection against mycobacteria. Epithelial cells participate in activation of innate immunity, as well as adaptive immunity, inducing the recruitment and activation of dendritic cells and T and B lymphocytes, which in turn increase antigen recognition and production of antibodies and other immune substances [58, 59]. These markers merit further investigation for the ability to distinguish early and late mycobacterial infection.

Despite some signals suggesting that the biomarker expression differences between LTBI and active TB can be used for diagnostics, choosing a panel of reproducible, discriminatory markers based on the results of the studies analyzed is quite difficult due to

1. failure to account for the time/illness of the individuals studied. Not surprisingly, the biology of TB is much more complex than previously thought, and therefore, classification in LTBI and active TB is insufficient. What is considered LTBI actually corresponds to a range of infection status, which may have been recently acquired or present for decades. Recently acquired TB is associated with a higher progression rate to active disease pointing to distinct biological properties. The study of pulmonary immune substances in the animal model reveals changes in the expression of cytokines/chemokines in the cells that make up the granuloma. The diversity of granulomas (diverse functions and architectures and microenvironments) has consequences on the bacterial control [60, 61]. It is suggested that after the in vitro stimulation, changes in the cellular expression due to the phase of infection or tuberculosis disease can lead to a varied response that is evidenced in the analyzed studies. Among the papers included, there were 22 different antigens used for in vitro stimulation, with concentrations that widely varied within the same immune factors and within the same group of patients; for example, INF-*γ* ranges from 0 to 2640 pg/ml, with overlapping concentrations between the uninfected individuals, LTBI, and active TB group, independent of the sample used (Additional file 6).
2. Given the heterogeneity of the biology of disease associated with LTBI, the ability to identify the duration of infection remains a challenge for future research. Inability to determine the duration and type of LTBI (i.e., what type of granuloma) might modify the observed response to a mycobacterial antigen leading to blurring of the ability to interpret differences between study groups [60, 61].
3. different samples and varied cell stimulation protocols. The samples used for the studies were predominantly plasma; however, culture supernatant, serum, and RNA were used introducing variability in measured concentration caused by the matrix used (Table 1 and Additional file 6). Several potential reasons for the variation in immune substance concentrations in plasma and serum from whole blood include inhibition of detection for specific cytokines (e.g., EGF, GM-CSF, IL-3, and IL-4) in the serum [62]; delay in processing of serum or plasma, sample hemolysis, presence of debris, or freeze-thaw cycles, all of which can adversely affect cytokine detection [63]; and the release of several mediators by platelets which can increase cytokine serum levels, especially CCL5 and CD40L [64].
4. In addition, the wide variety of antigens (ESAT-6, CFP-10, TB7.7, PPD, or Mtb CFA, among others) used to stimulate cells and different incubation times leads to the increases or decreases of the time of cellular exposure to the stimulus and therefore the concentration of the detected immune mediators and other substances. In addition, cellular stimulation adds complexity to the diagnostic utility of detecting biomarkers, especially in areas with limited laboratory infrastructure or access, as is the situation in many of the countries or settings where TB is endemic. In addition, reviewed articles show variations in the results due to the antigen used for stimulation [11–13]. In experiments with whole bacteria, it has been demonstrated that the strain used to carry out stimulation modifies the type of immune response in vitro; for example, the most recent strains in the *M. tuberculosis* lineage show a lower inflammatory response in macrophages when compared to the older strains [65]. Likewise, Leyten et al. evaluated 25 antigens of latency related to the DosR regulator of *M. tuberculosis*; it was observed that different antigens can give different cellular responses (measured by the production of INF-*γ*) after in vitro stimulation, and in addition, this can vary between healthy people, LTBI and active TB cases [11]. This limitation can be overcome in longitudinal studies applying the same measurement at different times along the natural history of *M. tuberculosis* infection.
5. the variety of laboratory methods used for detection of substances, which in turn leads to the variable units of measurement and assay sensitivity. The ability to compare the heterogeneous samples is further compounded by use of ELISA, microbead assays, EIA, and real-time PCR—in the absence of an endogenous standard—yielding variable dynamic ranges [66]. The intraindividual variability cannot be assessed, as only 2 studies were longitudinal. This variability results in difficulty to compare and quantify studies
6. the presence of a selection bias for nonapplication of the booster when individuals are screened using the tuberculin skin test. It is known that the booster effect can occur in individuals and is only detected when a second TST is applied to negative individuals between 1 and 4 weeks after the first administration. The increase in the frequency of positive individuals is notable in the population without any underlying diseases (in prisoners, an increase in positivity was reported from 66% to 77.6% [67]) or in those with other disorders such as rheumatoid arthritis (where the booster positivity changed from 31.3% and 21.7% to 46.5% and 28.8% in early and late rheumatoid arthritis, respectively) [68]. The lack of application of two-step TST may lead to erroneous classification to the uninfected group, resulting in false negative results [67–70]. Equally important, LTBI diagnoses were done using TST and/or IGRAs, which can introduce heterogeneity within the results. Indeed, in many studies when both methods were used, the results demonstrated inconsistent findings, a common theme discussed in literature [71–73]. Additionally, although some articles used TST for LTBI diagnosis, they did not consider the rate of BCG vaccination within children under 10 years old in their analyses [74]
7. the fact that none of the studies adjusted the analysis for the effect of ethnicity on the association between IP concentrations and the different stages of TB. A study published by Coussens et al. reported that the inflammatory profile differs according to ancestry. Individuals of African descent with TB, despite having similar mycobacterial strains and similar sociodemographic and clinical characteristics, have a different inflammatory profile compared to Eurasian patients with the same disease [75]. Similarly, Mwantembe et al. reported ethnic variation of cytokines (IL-1RA, IL-12) and chemokines (CCL2, CCL5, CCL11, and CXCL8) in South African patients with inflammatory bowel disease [76]. The concentrations of these chemokines and cytokines are determined by allelic frequency and have been involved in response to *M. tuberculosis* infection. Likewise, genes coding for proteins such as CCL2 [77], IL-17F, IL-17A [78], and IL-12 [37, 79] have been described as polymorphic; variation in allele frequency is affected by ethnic variation, affecting the antimycobacterial response, and thus may be driving the higher risk for development of TB among different populations. The examples emphasize the importance to adjust by ethnicity of the population at the time of reporting the results as these clearly impact biomarker concentrations.
8. Ethnicity is also related to the response to current *M. tuberculosis* infection screening tests. Genetic variants associated with the reaction to TST and IGRAs have been described. The TST1 locus is associated with a TST positivity per se (TST1 on 11p14), and the TST2 locus is associated with the intensity of TST reactivity (TST2 on 5p15) [3]. On the other hand, the production of INF-*γ* has been associated with genetic factors such as the locus located in chromosomal regions 8q12-22n and 3q13-22 [2]. The ethnicity must be considered when performing the immunological analysis in further research.
9. the heterogeneity of the populations studied. First, the comparison group of active TB included patients with pulmonary TB and extrapulmonary TB together. In these two groups of patients, the presentation of the disease is different, and the main factors of innate immunity, cytokines and chemokines, which play a role in cell-mediated immunity, involved in the dissemination of M. tuberculosis, differ. Mutations have been reported in genes encoding the INF-*γ* receptor, the IL-12 receptor, and the transcription-activating signal1 (STAT-1) in patients with extrapulmonary TB. Likewise, Yang et al. reported that there are differences in the immunopathogenicity of pulmonary and extrapulmonary infections. The production of CCL2, CXCL9, and CXCL8 modifies the type of tuberculous disease that a patient has, and they play a special role in the formation of granuloma [80]. Patients with pulmonary TB showed lower levels of the cytokines studied than those with extrapulmonary TB. CXCL8 concentration was found to be elevated in fatal TB, increases in CCL2 were observed with disseminated and meningeal TB [81], and TGF-*β* increased in extrapulmonary TB in children compared to pulmonary TB [14].
10. Secondly, patients with active TB included in the studies were at different phases of treatment (before, during, and after completion of antituberculous therapy). Several studies have been carried out with the aim of evaluating new biomarkers that allow monitoring the patient's condition after initiating antituberculous therapy. Several of the studies were longitudinal, making it evident that the immune substances changed during the administration of TB treatment [82, 83]. Changes in lung bacterial load related to treatment administration would appear to influence the concentration of cytokines detected in nonstimulated cells, with 17 out of the 27 cytokines/chemokines analyzed (IL-1*β*, IL-2, IL-4, IL-5, IL-6, IL-9, IL-13, IL-17, eotaxin, IFN-*γ*, IP-10, MCP-1, MIP-1*α*, MIP-1*β*, PDGF, RANTES, and VEGF) being significantly lower in patients with higher bacterial load and levels of IL10, IL15, and TNF-*α* being higher in the same patients [84].

## 5. Conclusions

Identification of biomarkers that individually or in combination can differentiate LTBI and active TB has been a research priority; however, a constellation of markers that differentiate between infection and disease is not yet available. The advances in high-throughput technologies for biomarker measurement are promising, but the variability of studies and potential biases that we have highlighted undermines the ability to identify reproducible markers. Although five parameters were exclusively increased in LTBI and 24 in active TB, only a single substance was consistently differential. These substances were not measured in all studies, and results are inconsistent between study groups, prohibiting the desired classification. Undoubtedly, the study of multiple immune substances seems to give better results than the study of a single biomarker; consequently, the search for immune profiles with multiple immune substances should be the goal of future research.

For the results obtained with different immune markers, future research should “harmonize” the methodological conditions to evaluate immune markers as the first step to draw any conclusion about LTBI parameter(s) for use as a diagnostic test. Those aspects include the presence of the booster effect, clinical classification of TB, the ethnicity of participants, and sample size estimation. In addition, cohort studies will allow identification of immune substances related to progression to active TB and conversion to LTBI and variations in the immune response due to the individual's stage of TB, measurement variation for cytokine/chemokines, and hormonal influences [85, 86].

The BCG has been associated with modulating the host's immune system and granting protection against MTB infection and disease [87, 88]. BCG vaccination could potentially modify the concentration of the immune substances in vaccinated adults, considering that it changes the concentrations in children and adolescents [89]. Further studies should evaluate the effect of BCG vaccination in the immune marker response.

It is important to note that our review did not include HIV-infected populations or any other types of immunosuppression, nor children, since those populations have several confounders and particular characteristics that need to be analyzed separately and are beyond the scope of the present review. In addition, as the main goal of our paper was to identify immune markers associated with LTBI, we did not include articles that consider biomarkers for TB before and after the treatment.

## Figures and Tables

**Table 1:** Characteristics of studies included in the systematic review.

First author, year of publication	Country where the study was conducted	Outcome of interest	Special feature of the population under study	Number of people with LTBI	Number of people in the control group	Age	Sex (% women)	Proportion with BCG vaccination (%)	Active TB diagnosis method	LTBI diagnosis method	Immune parameters evaluated	Increased immune parameters (with statistical differences)^∗^	Samples	Antigen and times used for stimulation	Method of detection of cytokines, commercial kit
Zeev T. Handzel, 2007 [23]	East European, Ethiopian and Israel	LTBI	Immigrant patients from Eastern Europe and Ethiopia and their contacts in Israel	39	PTB = 39 NI = 21	Not reported	Not reported	Not reported	Culture, clinical diagnosis, X-ray, TST > 15	TST	INF-*γ*, IL-2R, IL-10, IL-6, IL-12p70	Unstimulated LTBI vs. NI: IL-10 and IL-6 Stimulated LTBI vs. NI: INF-*γ* TB vs. LTBI: sIL-2R, INF-*γ*, and IL-10 Serum TB vs. LTBI: sIL-2R, IL-10	Plasma samples from whole blood (unstimulated, antigen-stimulated, or mitogen-stimulated) and serum	PPD Time: 48 hours	ELISA (R&D Systems, Minneapolis, MN, USA)

Novel N. Chegou, 2009 [24]	South Africa	LTBI	Contacts of people with TB and patients with TB from an endemic area	34	PTB = 23	Mean ± SD TB: 30.3 ± 13.6 LTBI/NI: 31.8 ± 14.2	TB: 26 LTBI/NI: 58.8	Not reported	Smear (ZN)	QuantiFERON-TB Gold In-Tube Test, TST	(IL)-1*α*, IL-1*β*, IL-2, IL-4, IL-5, IL-6, IL-7, IL-10, IL-12(p40), IL-12(p70), IL-13, IL-15, IL-17, CXCL8, IL-1ra, sCD40L, CCL11, fractalkine, G-CSF, GM-CSF, IFN-*γ*, CXCL10, CCL2, CCL3, CCL4, TGF-*α*, TNF-*α*, VEGF	Unstimulated TB vs. LTBI: EGF, TGF-*α*, TNF-*α*, and sCD40L Stimulated LTBI vs. TB: sCD40L, VEGF. TB vs. LTBI: IL-1*α*	Plasma samples from whole blood (unstimulated, antigen-stimulated, or mitogen-stimulated)	ESAT-6, CFP-10, and TB7.7 Time: not specifically referred	Microbead-based method, LINCO-plex® kits (Millipore, St. Charles, Missouri, USA)

R. Biselli, 2010 [25]	Italy	LTBI	Laboratory personnel without *M. tuberculosis* infection and TB cases of infectious diseases L. Spallanzani, and the Infectious Diseases Department of Sapienza Universita di Roma	20	PTB = 20 NI = 20	Median LTBI: 42.3 TB: 35.7 NI: 31.4	LTBI: 40 TB: 45 NI: 30	LTBI: 0 TB: 35 NI: 0	Culture	QuantiFERON-TB Gold In-Tube Test, TST	INF-*γ*, IL-2	Stimulated LTBI and TB vs. NI: INF-*γ* LTBI vs. TB and NI: IL-2	Plasma samples from whole blood (unstimulated, antigen-stimulated, or mitogen-stimulated)	ESAT-6, CFP-10, and TB7.7 Time: 18 and 72 hours	ELISA, ELISA assay (DRG GmbH, Germany)

Jayne S. Sutherland, 2010 [26]	South Africa	LTBI	TB case contacts and TB cases	20	TB/NRCF = 36 NI = 19	Median (IQR) LTBI: 27 (19–39) TB: 25 (20–37) NI: 22 (18–31)	TB: 27 LTBI: 74 NI: 65	Not reported	Smear (ZN, auramine-rodhamine), culture	TST	TNF-*α*, IFN-*γ*, IL-10, IL-12(p40), IL-13, IL-17, IL-18	Stimulated LTBI vs. NI: IFN-*γ*, IL-13, and IL-17 TB vs. NI: IL-10, IL-12(p40), IL-13, IL-17, IFN-*γ*, and TNF-*α*. TB vs. LTBI: TNF-*α* and IL-12(p40)	Blood culture supernatant unstimulated or antigen-stimulated	ESAT-6/CFP-10, PPD, or TB10.4 Time: 7 days	Microbead-based method, 7-plex kit, BioRad

Subash Babu, 2010 [27]	India	LTBI	Adult population with and without *M. tuberculosis* exposure	25	NI = 25	Median (range) LTBI: 32 (19-50) NI: 30 (15-48)	LTBI: 40 NI: 40	All participants	N/A	TST	IL-2, IFN-*γ*, TNF-*α*, IL-12, IL-4, IL-5, IL-10, IL-13, IL-17, IL-23, IL-6, IL-1*β*, IL-23	Stimulated NI vs. LTBI: IL-17, IL-23	Nonstimulated and antigen-stimulated PBMC culture supernatants	PPD or Mtb CFA Time: 24 hours	Microbead-based method and ELISA for IL-23, BioRad

Marc Frahm, 2011 [28]	Not reported	LTBI	Adult population with and without TB from two previous cohorts	32	PTB = 9 EPTB: 3 NI = 26	Median (range) LTBI: 50 (2–66) TB: 43.5 (4–93) NI: 46.5 (26–62)	LTBI: 47 TB: 33 NI: 27	LTBI: 31 TB: 33 NI: 0	Culture from a clinical specimen or clinical diagnosis	QuantiFERON-TB Gold In-Tube Test, TST	IL-1*β*, IL-1RA, IL-2, IL-2R, IL-4, IL-5, IL-6, IL-7, IL-8, IL-10, IL-12 p40/70, IL-13, IL-15, IL-17, TNF-*α*, IFN-*α*, IFN-*γ*, GM-CSF, MIP-1*α*, MIP-1*β*, IP-10, MIG, eotaxin, RANTES, MCP	Stimulated LTBI and TB vs. NI: INF-*γ*, IP-10, MIG, IL-2, MCP-1, IL-15, IL-RA. TB vs. LTBI: IL-15. With a more flexible cut-off point: MCP-1, IL-1RA, IFN-*α* and IL-4	Plasma samples from whole blood (unstimulated, antigen-stimulated, or mitogen-stimulated)	ESAT-6, CFP-10, and TB7.7 Time: 16–24 hours	Microbead-based method, Human Cytokine 25-plex (Biosource, Camarillo, CA)

Ji Young Hong, 2012 [46]	Korea	LTBI	Contacts of patients with confirmed TB. Cases of TB were hospitalized patients with comorbidities	22	PTB = 46 NI = 32 Combined EPTB lesion: 2 (4.3%)	Median (range) LTBI: 37.5 (22–53) TB: 30 (22–74) NI: 28 (22–57)	LTBI: 18 TB: 25 NI: 18	LTBI: 90.9 TB: 54.3 NI: 75.0	Culture	QuantiFERON-TB Gold In-Tube Test, TST	IP-10, INF-*γ*	Unstimulated plasma TB vs. LTBI and NI: IP-10 Stimulated plasma LTBI and TB vs. NI: IP-10, INF-*γ* Serum TB vs. LTBI and NI: IP-10	Plasma samples from whole blood (unstimulated, antigen-stimulated, or mitogen-stimulated) and serum	ESAT-6, CFP-10, and TB7.7 Time: 20 hours	ELISA (R&D Systems, Minneapolis, MN, USA)

S.Y. Kim, 2012 [45]	Not reported	LTBI	TB case partners with and without LTBI	19	PTB = 32 NI = 30	Median (range) LTBI: 47 (23-60) TB: 31 (20-77) NI: 28 (22-57)	LTBI: 68.4 TB: 46.8 NI: 53.3	LTBI: 94.7 TB: 64.5 NI: 76.7	Smear (ZN), culture, and/or pathology	QuantiFERON-TB Gold In-Tube Test, TST	IFN-*γ*, IL-2, IL-10, IL-13, IL-17, TNF-*α*	Stimulated LTBI and TB vs. NI: IFN-*γ*, IL-2, IL-10, and IL-13	Plasma samples from whole blood (unstimulated, antigen-stimulated, or mitogen-stimulated)	ESAT-6, CFP-10, and TB7.7 Time: 20 hours	Microbead-based method, MILLIPLEX® MAP human cytokine/chemokine kit (Millipore, Billerica, MA, USA)

Pierre-Alain Rubbo, 2012 [29]	France	LTBI	Healthcare workers with high risk of *M. tuberculosis* exposure	41	NI = 29	Median (IQ): 44 (36–50)	All participants: 84.3	All participants	N/A	QuantiFERON-TB Gold In-Tube Test	IL-1RA, IL-2, IL-2R, IL-4, IL-5, IL-6, IL-7, IL-10, IL-12p40/70, IL-13, IL-15, IL-17, TNF-*α*, GM-CSF, MIP-1*α*, MIP-1*β*, IP-10, MIG, eotaxin, RANTES, MCP, IFN-*γ*	Stimulated LTBI vs. NI: IL-2, IL-15, IP-10, and CXCL9	Plasma samples from whole blood (unstimulated, antigen-stimulated, or mitogen-stimulated)	ESAT-6, CFP-10, and TB7.7 Time: 24 hours	Microbead-based method, cytokine human panel (Invitrogen, Villebon sur Yvette, France)

Sen Wang, 2012 [30]	China	LTBI	Adults living in an endemic area to TB	73	PTB = 66 NI = 76	Median (range) LTBI: 41 (18–83) TB: 45 (16–86) NI: 38 (18–50)	LTBI: 52.1 TB: 40.9 NI: 45.2	LTBI: 74.0 TB: 78.9 NI: 89.5	TB contact history, smear (ZN), culture, clinical diagnosis, and R-rays	QuantiFERON-TB Gold In-Tube Test, TST	IP-10, IL-2, TNF-*α*, INF-*γ*	Unstimulated LTBI vs. TB: IP-10 LTBI and TB vs. NI: IP-10, IL-2, TNF-*α*, INF-*γ* TB vs. LTBI: TNF-*α* Stimulated TB vs. LTBI: IFN-*γ*, IP-10, and IL-2	Plasma samples from whole blood (unstimulated, antigen-stimulated, or mitogen-stimulated)	ESAT-6, CFP-10, and TB7.7 Time: 20 hours	DuoSet ELISA, the DuoSet ELISA development kit (R&D Systems Inc, MN, USA)

Yang Yu, 2012 [31]	China	LTBI	Individuals exposed to *M. tuberculosis*, healthy volunteers without infection, and hospitalized patients with TB	20	PTB = 12 NI = 12	Mean LTBI 1: 40.7 LTBI 2: 46.1 TB: 38.5 NI: 30.7	LTBI 1: 60 LTBI 2: 50 TB: 58.3 NI: 41.6	Not reported	Culture, clinical diagnosis, X-ray, and/or HRCT	T–SPOT®, TST	CCL1, CCL2, CCL3, CCL4, CCL5, CCL7, CCL8, CCL11, CCL13, CCL15, CCL17, CCL20, CCL21, CCL24, CCL26, CCL27, CXCL5, CXCL, CXCL8, CXCL9, CXCL10, CXCL11, CXCL12, CXCL1, IL-2, IL-15, IL-4, IL-13, IL-7, IL-9, IL-5, GM-CSF, IL-6, IL-12, G-CSF, TNF-*α*, IL-10, IFN-*γ*, IL-1RA, IL-1*β*, IL-17	Stimulated PBMCs LTBI 1 vs. NI: IP-10, CXCL11, and CXCL12 LTBI 2 vs. LTBI 1: IL-2, CXCL10, CXCL11, and CXCL12 TB vs. NI: IL-2, IP-10, CXCL11, IL-6, IL-9, IL-10, CCL-8, CXCL13, CXCL12, CCL1, CCL21 Plasma: TB vs. NI: IL-6, CCL1, IL-9, and CXCL9	Nonstimulated and antigen-stimulated PBMC culture supernatants and plasma	Lysed bacteria proteins and ESAT-6 Time: 72 hours	Microbead-based method, human cytokine/chemokine panel (MPXHCYTO-60K, MPXHCYP2-62K, and MPXHCYP3-63K, Millipore, USA)

Novel N. Chegou, 2012 [32]	South Africa	LTBI	TB case contacts and TB cases from a high TB-endemic community	23	PTB: 15	Mean (SD) 31.5 (15.9)	All participants: 39.5	Not reported	ZN	TST	EGF, fractalkine, IFN-a2, IFN-c, IL-4, IL-10, IL-12(p40), TGF-a, TNF-a, VEGF, IP-10, RANTES	Unstimulated TB vs. contact: EGF, IFN-a2, and IL-4. Stimulated ESAT-6/CFP-10 TB vs. contacts: EGF, TGF-a, and TNF-a. Stimulated Rv0081 Contacs vs. TB: IFN-g, IFN-a2, IL-12(p40), IP-10, TNF-a, VEGF, IL-10, and RANTES. Stimulated Rv2032 TB vs. contacts: fractalkine, IL-12(p40), TGF-a, TNF-a, VEGF, IL-10, RANTES. Stimulated Rv1737c TB vs. contacts: IL-10, TGF-a, TNF-a, IL-12(p40), and EGF	Plasma samples from whole blood (unstimulated or antigen-stimulated)	Resuscitation-promoting factors (Rv0867c, Rv2389c) and DosR regulon-encoded antigens (Rv2032, Rv0081, Rv1737c) Time: 7 days	Microbead-based method, Milliplex kits (Merck Millipore, St. Charles, Missouri, USA)

D. Anbarasu, 2013 [33]	India	LTBI	Family of TB cases from an endemic area to *M. tuberculosis*	7	PTB = 10	Range LTBI: 28-55 TB: 26-52	LTBI: 28.6 TB: 30	Not reported	Smear (ZN) and culture	TST	IL-1*β*, IL-1RA, IL-2, IL-5, IL-6, IL-7, IL-8, IL-9, IL-10, IL-12 (p70), IL-13, IL-15, IL-17, eotaxin, FGF basic, G-CSF, GM-CSF, IP-10, MCP-1, MIP-1*α*, MIP-1*β*, PDGF, RANTES, and VEGF	Stimulated CFP-10, Rv3716c, and TrxC LTBI vs. TB: IL-6 Stimulated FbpB/Rv2626c TB vs. LTBI: G-CSF, IL-7, IL-8, IL-9, and PDGF. LTBI vs. TB: IL-6	Blood culture supernatant unstimulated or antigen-stimulated	Protein fraction 11_24 (Rv2626c and FbpB) Time: 6 days	Microbead-based method, Bio-Plex multiplex cytokine assay system (Bio-Rad Laboratories, Hercules, CA, USA)

Yun-Gyoung Hur, 2013 [47]	Malawi	LTBI	TB cases from a cohort with their contacts	It is not clear (143 in LTBI and NI)	PTB = 15	Mean TB: 41 LTBI: 40	LTBI: 67 TB: 60	Not reported	Smear (ZN)	TST	IL-10, IL-13, IL-17, CXCL10, TNF-*α*.	Stimulated LTBI and TB vs. NI: IFN-*γ*, CXCL10, IL-10, TNF-*α*, and IL-17. LTBI vs. TB and NI: IL-10 LTBI vs. TB: IL-17 TB vs. LTBI: IL-17 and IL-10, in the following	Blood culture supernatant unstimulated or antigen-stimulated	PPD or ESAT-6 Time: 6 days	DuoSet ELISA, R&D Systems

Mayer-Barber, 2014 [34]	China and India (cohort reported by Andrade BB, 2013)	LTBI	TB cases from a Chinese cohort and healthy community controls India TB cases (pulmonary and extrapulmonary), LTBI, and healthy donors recruited as part of a TB cohort study	China: 14 *India*: 39	PTB = 94 Healthy controls = 11 *India*: PTB: 97 EPTB: 35 Healthy controls: 40	Median (IQR) PTB: 27 (23-44.7) Healthy controls: 33 (23-40) LTBI: 38.5 (34.2-43.5) *India* Median (IQR) Healthy control: 29 (21-59) LTBI: 25 (21-49) EPTB: 33 (18-65) PTB: 40 (19-70)	PTB: 38.3 Healthy controls: 54.5 LTBI: 85.7 *India* PTB: 33 EPTB: 84 Healthy controls: 75 LTBI: 77	Not reported	Smear (ZN) *India* Smear and culture	QuantiFERON-TB Gold In-Tube Test *India* QuantiFERON-TB Gold In-Tube Test and TST, absence of chest radiograph or pulmonary symptoms	IL-1*α*, IL-1*β*, IL-10, IL-1Ra, IL1R1, IL1R2, IFN-*γ*, IFN-*α*, IFN-*β*, TNF-*α*, PGF2*α*, PGE2, LXA4, 15-Epi-LXA4 *India* IL-1*α*, IL-1*β*, IL-10, IL-1Ra, IFN-*γ*, IFN-*α*, IFN-*β*, TNF-*α*, PGF2*α*, PGE2, LXA4, 15-Epi-LXA4, IL-1R1, IL-1R2	LTBI vs. NI and TB IFN-*α* NI vs. LTBI and TB: IL-1*α*, IL-1*β*, TNF-*α*, IL1Ra TB vs. LTBI and NI: IL-10, IL-1RI, IFN-*γ*, PGF2*α*, PGE2 *India* LTBI vs. NI and TB: IL1Ra, PGF2*α* NI vs. LTBI and TB: IL-1*α*, sIL-1R1 TB vs. LTBI and NI: IL-1 *β*, PGE2, TNF-*α*, IFN-*γ*, IFN-*α*, IL-10, LXA4, 15-Epi-LXA4	Plasma samples	Not apply	ELISA kits (R&D Systems) and FlowCytomix Multiplex Arrays (eBioscience, San Diego, CA) and Oxford Biomedical Research (Oxford, MI) *India* ELISA kits (R&D Systems) and enzyme immunoassay (EIA) kits (Cayman Chemical, Ann Harbour, MI) and Oxford Biomedical Research (Oxford, MI)

Ikaria Sauzullo, 2014 [35]	Italy	LTBI	Healthcare workers studied for LTBI	TST+/QFT− = 34 TST+/QFT+ = 29 Total 63	PNI = 126	Mean (range) 43 (25–60)	All participants: 50.5	All participants: 3.1	N/A	QuantiFERON-TB Gold In-Tube Test or TST and had one of the following risk factors: chest X-ray suggestive of prior TB infection, a history of exposure to a case of active TB, or coming from an area with a high prevalence of TB infection	IFN-*γ*, IL-2	Stimulated LTBI vs. NI: IL-2, INF-*γ*	Plasma samples from whole blood (unstimulated, antigen-stimulated, or mitogen-stimulated)	ESAT-6, CFP-10, and TB7.7 Time: 72 hours	ELISA (DRG GmbH, Germany)

K. Kim, 2014 [36]	Australia	LTBI	Patients of the Western Australian Tuberculosis Control Program	30	PTB = 23 EPTB: ~8 (25%)	Median (IQR) LTBI: 32 (25-39) TB: 35 (29-42.5)	LTBI: 50 TB: 32.3	Not reported	Culture	IGRAs, TST	IFN-*γ*, TNF-*α*, IL-10	Stimulated LTBI vs. TB: IFN-*γ* TB vs. LTBI: TNF-*α*	Nonstimulated and antigen-stimulated PBMC culture supernatants	PPD, ESAT-6, or CFP-10 Time: 6 hours	ELISA, BD OptEIA™ Sets (BD Biosciences, USA)

Yun Hee Jeong, 2015 [37]	South Korea	LTBI	Patients with active TB and contacts with LTBI	20	PTB: 33 NI: 26	Median (range) LTBI: 44 (22–60) TB: 30 (20–63) NI: 25 (22–54)	LTBI: 80 TB: 38.7 NI: 53.8	LTBI: 90 TB: 63.6 NI:5 3.8	Clinical, radiological, microbiological, and/or pathological results	TST	IL-2, IL-6, IL-8, IL-10, IL-13, TNF-*α*, IFN-*γ*, MIG, IP-10, I-TAG, and MCP-1	Unstimulated LTBI vs. TB: IL-2, IL-10, IL-13, IL-8, and IFN-*γ* Stimulated TB vs. NI: IL-2, IL-6, IL-13, MIG, IP-10, I-TAG, MCP-1, and IL-8. TB vs. LTBI: IL-2, IL-6, IL-10, IL-13, TNF-*α*, MIG, IP-10, I-TAG, INF-*γ* LTBI vs. NI: IL-8	Plasma samples from whole blood (unstimulated, antigen-stimulated, or mitogen-stimulated)	ESAT-6, CFP-10, and TB7.7 Time: 24 hours	Microbead-based method, BD Biosciences, San Jose, CA, USA

Babak Pourakbari, 2015 [48]	Iran	LTBI	Individuals vaccinated and without previous exposure to *M. tuberculosis* and patients infected with *M. tuberculosis*, taken at the hospital	30	PTB = 30 NI = 30	Mean ± SD LTBI: 40.2 ± 15.8 TB: 35.3 ± 18.8 NI: 45.3 ± 5.6	LTBI: 27 TB: 13 NI: 73	Not reported	Culture	QuantiFERON-TB Gold In-Tube Test, TST	IL-2	Stimulated LTBI vs. TB and NI: IL-2	Plasma samples from whole blood (unstimulated, antigen-stimulated, or mitogen-stimulated)	PE35 (Rv3872) and PPE68 (Rv3873) Time: 3 days	ELISA, ELISA kit (Mabtech AB, Sweden)

Prachi R. Bapat, 2015 [22]	India	LTBI	Individuals living with TB cases and individuals in the community. Malnourished population	QFT+/TST+ = 26 QFT+/TST− = 12 QFT−/TST+ = 1 Total 39	NI = 35 Community = 16	Mean (range) 34.4 (12-65)	All participants: 45.9	All participants: 30	N/A	QuantiFERON-TB Gold In-Tube Test, TST	IL-6, IL-10, IL-2, TNF-*α*R, INF-*γ*	Stimulated LTBI vs. NI: IL-6 LTBI and NI vs. community: IL-6, IL-10 NI vs. LTBI: IL-10	Plasma samples from whole blood (unstimulated or antigen-stimulated)	ESAT-6, CFP-10, and/or TB7.7 Time: 20–24 hours	Microbead-based method. IMMULITE-1000 Immunoassay System (Siemens Healthcare Global)

Yun-Gyoung Hur, 2014 [49]	Korea	LTBI	Adults with TB, individuals recently exposed to *M. tuberculosis*, healthy participants without *M. tuberculosis* exposure, and patients with non-TB mycobacteria infections	51	PTB = 86 NI = 133 EPTB: 1 (1.7%)	Median (range) LTBI: 44 (18-82) TB: 32 (20-76) NI: 31 (20-61) MNT: (43-84)	LTBI: 74.5 TB: 49 NI: 51 MNT: 76.1	LTBI: 84.6 TB: 56.9 NI: 63.6 MNT: 60.5	Smear/culture or R-rays	QuantiFERON-TB Gold In-Tube Test, TST	IL-1*β*, IL-2, IL-4, IL-5, IL-6, IL-9, IL-10, IL-12p70, IL-13, IL-17A, IL-22, IFN-*γ*, TNF-*α*, IFN-*α*, sCD40L, CXCL10, VEGF-A	Stimulated TB and LTBI vs. controls: IFN-*γ*, IL-2, CXCL10 Serum TB vs. NI: IL-22, CXCL10, and VEGF-A. TB vs. LTBI: VEGF-A TB vs. MNT: IL-2, IL-9, IL-13, IL-17, and TNF-*α* MNT vs. TB: sCD40L	Plasma samples from whole blood (unstimulated, antigen-stimulated, or mitogen-stimulated) and serum	ESAT-6, CFP-10, and TB7.7 Time: 24 hours	Microbead-based method, BD FACSVerse (BD Biosciences, San Jose, CA, USA)

M. Wei, 2015 [38]	China	LTBI	Controls hospitalized for other causes without radiological signs of TB and patients hospitalized for TB	40	PTB = 40 NI = 40	Mean ± SD LTBI: 18.0 ± 10.35 TB: 18.47 ± 12.68 NI: 16 ± 9.06	LTBI: 55 TB: 47.5 NI: 50	Not reported	Clinical diagnosis	T–SPOT®, TST	CCL1, CXCL9, IL-6, IL-10, CSF3, CSF2, IL-1*α*, IL-8, IL-7, IL-2, TGF-*β*1, CCL2, TNF-*α*	Unstimulated TB vs. LTBI and NI: CCL1, CXCL9, IL6, IL-10, CSF3, CSF2, IL-1-*α*, IL-8, IL-7, IL-2, TGF-*β*1, CCL2, TNF-*α*. Stimulated TB vs. LTBI: CCL1 (I-309), CXCL9 (MIG), IL-10, IL-6, CSF2, CSF3, IL-8, IL-1*α*, IL-7, TGF-*β*1, CCL2, IL-2, and IL-13	Plasma samples from whole blood (unstimulated, antigen-stimulated, or mitogen-stimulated)	ESAT-6 and CFP-10 Time: 20 hours	Quantitative immunomicroarray (Quantibody Human Cytokine Array 1, RayBiotech, Inc., Norcross, GA)

Ji Yeon Lee, 2015 [39]	Korea	LTBI	Healthy and TB patients from the National Medical Center and Community Health Center of Korea	25	PTB = 24	Mean (range) LTBI: 48 (23-59) TB: 48 (28-75)	LTBI: 44 TB: 37.5	Not reported	Smear (ZN) and/or cultures and X-rays	TST	IL-1, IL-6, IL-10, TNF-*α*, IL-17, GM-CSF, IL-4, IL-1*β*, INF-*γ*, LXA4, and PGE2	Monocyte stimulated MTSA LTBI vs. TB IL-10 MTSA+INF-*γ*: IL-1, IL-6, IL-10 Stimulated CD4+ T cells and monocytes with PPD: TNF-*α*. Plasma TB vs. LTBI: LXA4 and PGE2	Nonstimulated and antigen-stimulated PBMC culture supernatants and plasma samples	H37Rv soluble antigens Time: 5 days	Microbead-based method (Bio-Rad Laboratories, Hercules, CA) ELISA for IL-1*β*, ELISA kit (R&D Systems) EIA for LXA4 (Oxford Biomedical Research, Oxford, MI) EIA for PGE2 (Cayman Chemical, Ann Arbor, MI) Bio-Plex Multiplex Immunoassay Systems (Bio-Rad Laboratories, Hercules, CA)

Mulugeta Belay, 2015 [21]	Ethiopia	LTBI	Individuals from health centers in an endemic area to TB	148	PTB = 147 NI = 68	Mean LTBI: 32 TB: 29.4 NI: 32.4	LTBI: 55.5 TB: 41.5 NI: 52.9	LTBI: 37 TB: 28.1 NI: 35.3	Smear (ZN)	QuantiFERON-TB Gold In-Tube Test	IFN-*γ*, TNF-*α*, IL-10	Stimulated Basal: NI vs. LTBI and TB: IFN-*γ*, TNF-*α*, IL-10 NI and TB vs. LTBI: IFN-*γ*, TNF-*α*, and IL-10 Six months: TB and LTBI vs. NI: INF-*γ*. TB and LTBI TNF-*α* and IL-10: baseline < 6 months < 12 months	Blood culture supernatant unstimulated or antigen-stimulated	E6C10 and Rv2031 Time: 48 hours	ELISA, Ready-Set-Go! cytokine ELISA kits (eBioscience, USA)

Sunghyun Kim, 2015 [50]	Korea	LTBI	Adult population, contacts of TB cases with and without *M. tuberculosis* infection	22	PTB = 28 NI = 29	Mean (range) LTBI: 46.5 (22-69) TB: 32.1 (21-69) NI: 30.1 (22-44)	LTBI: 86.3 TB: 71.4 NI: 79.3	LTBI: 95.5 TB: 32.1 NI: 79.3	Culture	QuantiFERON-TB Gold In-Tube Test, TST	IFN-*γ*, TNF-*α*, IL-2R, IL-4, IL-10, CXCL9, CXCL10, CXCL11	Stimulated LTBI vs. NI: IFN-*γ*, TNF-*α*, IL-2R, CXCL9, CXCL10 LTBI vs. TB: IL-17 TB vs. NI: INF-*γ*, TNF-*α*, IL-2R, CXCL9, CXCL10 TB vs. LTBI: TNF-*α*, CXCL11	RNA from antigen-stimulated whole blood cell pellets	ESAT-6, CFP-10, and TB7.7 Time: 24 hours	Real-time RT-PCR, TaqMan probe assay, and the ABI 7500 FAST instrument system (Applied Biosystems, Foster City, CA) ELISA

Ida Wergeland, 2016 [51]	Norway	LTBI	TB case and people with LTBI from a hospital	48	PTB = 14 EPTB: 4 NI = 16	Median (range) TB: 32 (18–62) LTBI: 40 (13–67) LTBI borderline: 40 (25–53) NI: 47 (16–68)	TB: 66.6 LTBI: 63.8 LTBI borderline: 63.6 NI: 75	Not reported	Culture or clinical diagnosis and X-ray	QuantiFERON-TB Gold In-Tube Test	IL-1*β*, IL-1, IL-1ra, IL-2, IL-4, IL-5, IL-6, IL-7, IL-8, IL-9, IL-10, IL-12 (p70), IL-13, IL-15, IL-17, basic FGF, eotaxin, G-CSF, GM-CSF, IFN-*γ*, IP-10, MCP-1, MIP-1*α*, MIP-1*β*, PDGF-BB, RANTES, TNF-*α*, and VEGF	Unstimulated LTBI vs. TB: IL-1*β*, IL-1ra, IL-9, and IL-17A. LTBI and TB vs. NI: RANTES NI vs. TB and LTBI: IL-15, eotaxin, and basic FGF NI vs. TB: IL-2, IL-4, IL-13, IL-17A, and IFN-*γ*. Stimulated TB and LTBI vs. NI: IL-1ra, IL-2, IL-13, IL-15, IFN-*γ*, IP-10, and MCP-1. LTBI vs. LTBI borderline and NI: IL-1ra, IL-2, IFN-y LTBI vs. NI: IP-10, IL-13, IL-15, IL-17A, MCP-1	Plasma samples from whole blood (unstimulated, antigen-stimulated, or mitogen-stimulated)	ESAT-6, CFP-10, and TB 7.7 Time: 16–24 hours	Microbead-based method, Bio-Plex Pro Human Cytokine Group 27-Plex Panel (Bio-Rad Laboratories Inc., Hercules, CA)

Tao Chen, 2016 [52]	China	LTBI	TB case and LTBI medical staff who worked at the institute for TB prevention. People with cancer and pneumonia	21	It is not clear	Mean ± SEM NI: 25.5 ± 9.1 LTBI: 38.0 ± 10.4 TB: 32.5 ± 12.7 Others: 48.6 ± 22.1	21	Not reported	Cough with blood-tinged sputum; fever; chest X-rays positive; microbiological test, IGRA positive	T–SPOT®, TST	IL-8, MIG, I-309, eotaxin-2, and ICAM-1	TB vs. NI and LTBI: IL-8, MIG, and I-309 LTBI vs. NI and others: eotaxin-2, ICAM-1, and MIG	Serum	N/A	Microarray and quantitative ELISA, Quantibody Human Cytokine Array 1, RayBiotech, Inc., Norcross, GA

Fabiana A. Zambuzi, 2016 [40]	Brazil	LTBI	TB case and people with LTBI from a hospital	14	PTB = 17 NI = 16	Mean LTBI: 31.4 TB: 39.6 NI: 27	LTBI: 78.6% TB: 17.6 NI: 81.2	Not reported	Microbiology confirmed and clinical diagnosis or X-ray	TST	IL-1b, IL-4, IL-5, IL-6, IL-10, IL-12p70, IFN-a2, TNF-a, IFN-*γ*, IP-10, RANTES, MCP-1, GM-CSF, IL-17, MIP-1a, MIP-1b, sCD163, and sCD14	TB vs. NI and LTBI: IL-6, IP-10, TNF-a, sCD163, and sCD14. LTBI vs. TB and NI: RANTES TB vs. LTBI: GMCSF	Plasma	N/A	DuoSet ELISA for sCD163 and sCD14 and microbead-based method, 16-plex, EMD Millipore Corporation, Billerica, Massachusetts, USA

Miguel Santin, 2016 [41]	Spain	LTBI	Adult population recruited at eight TB centers	43	PTB = 37 EPTB: 32 (46.4%) NI = 28	Median LTBI: 54 (46-64) TB: 41 (31-52) NI: 57 (44.5-77.3) Discordant: 49 (44.5-54)	Not reported	LTBI: 100 TB: 36.8 NI: 33.3 Discordant: 85.7	Microbiology confirmed or compatible when clinical, radiological, and/or ADA and/or histology positive, and cure was achieved after therapy	QuantiFERON-TB Gold In-Tube Test, TST	IFN-*γ*, IL-2	Stimulated LTBI vs. NI: IL-2, INF-*γ*	Plasma samples from whole blood (unstimulated, antigen-stimulated, or mitogen-stimulated)	ESAT-6, CFP-10, and TB7.7 Time: 72 hours	Quantitative ELISA, Quantikine® ELISA Human IL-2 Immunoassay (R&D Systems Inc., Minneapolis, MN, USA)

Xiangyang Yao, 2017 [42]	China	LTBI	Two cohorts each one with healthcare workers with LTBI and TB case	10 and 15	PTB = 40 and 20 NI = 9 and 15	Median (range) TB: 34.5 (20-78) and 29 (16-67) LTBI: 38.5 (20-48) and 38 (20-67) NI: 33 (18-56) and 48 (18-68)	TB: 60 and 45 LTBI: 60 and 60 NI: 44 and 35	TB: 35 and 25.8 LTBI: 100 and 100 NI: 100 and 86.7	Clinical, radiological, microbiological, and histopathological	QuantiFERON-TB Gold In-Tube Test	sCD40L, EGF, eotaxin, FGF-2, Flt-3 ligand, fractalkine, G-CSF, GM-CSF, GRO, IFN-*α*2, IFN-*γ*, IL-1*α*, IL-1*β*, IL-1ra, IL-2, IL-3, IL-4, IL-5, IL-6, IL-7, IL-8, IL-9, IL-10, IL-12 (p40), IL-12 (p70), IL-13, IL-15, IL-17, IP-10, MCP-1, MCP-3, MDC, MIP-1*α*, MIP-1*β*, TGF-*α*, TNF-*α*, TNF-*β*, VEGF 6Ckine, BCA-1, CTACK, ENA-78, eotaxin-2, eotaxin-3, I-309, IL-16, IL-20, IL-21, IL-23, IL-28A, IL-33, LIF, MCP-2, MCP-4, MIP-1d, SCF,SDF-1A+*β*, TARC, TPO, TRAIL, TSLP GCP2, I-TAC, IL-11, IL-29, lymphotactin, M-CSF, MIG, MIP-3*α*, MIP-3*β*	Unstimulated TB vs. NI and LTBI: sIL-2Ra, IP-10, and MIP-1a TB and NI vs. LTBI: IL-8 Stimulated TB and LTBI vs. NI: G-CSF, GM-CSF, IFN-*γ*, IL-1a, IL-2, IP-10, BCA-1, and eotaxin-2. TB vs. LTBI: G-CSF. TB vs. LTBI and NI: IL-8, VEGF, MCP-3	Plasma samples from whole blood (unstimulated, antigen-stimulated, or mitogen-stimulated)	ESAT-6 and CFP-10 Time: 22 ± 2 hours	Microbead-based method, Millipore Milliplex map system (EMD Millipore Corporation, Billerica, MA, USA)

Jing Wu, 2016 [53]	Not reported	LTBI	Contacts of TB cases with and without LTBI	36	PTB = 25 NI = 31	Mean (range) LTBI: 48 (7-76) TB: 51 (22-85) NI: 42 (5-80)	LTBI: 66.9 TB: 28 NI: 65.5	LTBI: 86.1 TB: 68 NI: 77.4	TB contact history, smear (ZN), culture, clinical diagnosis	T–SPOT®, TST	IL-1*β*, IL-1, IL-2, IL-4, IL-5, IL-6, IL-7, IL-8, IL-9, IL-10, IL-12(p70), IL-13, IL-15, IL-17, eotaxin, FGF, G-CSF, GM-CSF, IFN-*γ*, IP-10, MCP-1, MIP-1*α*, PDGF-BB, MIP-1*β*, RANTES, TNF-*α*, VEGF	Unstimulated LTBI vs. TB: IP-10, PDGF-BB, and RANTES. TB vs. LTBI: VEGF Stimulated LTBI vs. TB: IL-2, IL-10, IFN-*γ*, IP-10, and TNF-*α*. TB vs. NI: IL-2, IL-10, IP-10	Nonstimulated and antigen-stimulated PBMC culture supernatants	PPD Time: 24 hours	Microbead-based method, Bio-Plex Pro Human Cytokine 27-plex Assay (Bio-Rad, CA, USA)

R. Kamakia, 2017 [54]	Kenya	LTBI	Patients with suspected active TB and patients with active TB from Mbagathi District Hospital, Kenya, as well as contacts of people with TB	16	PTB = 19 NI = 8	Mean (IQR) LTBI: 35.6 (27-39.8) TB: 36.8 (25.8-5.15) NI: 33.5 (23.3-45.3)	LTBI: 50 TB: 21.1 NI: 75	Not reported	ZN, X-ray	QuantiFERON-TB Gold In-Tube Test	IL-17F, IFN-*γ*, GM-CSF, IL-10, IL-12p70, IL-13, IL-15, IL-17A, IL-22, IL-9, IL-1b, IL-33, IL-2, IL-4, IL-21, IL-23, IL-5, IL-6, IL-17E/IL-25, IL-27, IL-31, MIP-3*α*, TNF-*α*, TNF-*β*, IL-28A	Stimulated LTBI vs. TB: IL-17F, MIP-3*α*, IL-13, IL-17A, IL-5, INF-*γ*, IL-9, IL-2. LTBI vs. NI: INF-*γ*, IL-9, and IL-2	Plasma samples from whole blood (unstimulated, antigen-stimulated, or mitogen-stimulated)	ESAT-6, CFP-10, and TB7.7 Time: 2 hours	Microbead-based method, Milliplex MAP Human Th17 Magnetic Bead Kit (Millipore, St. Louis, MO, USA)

Eun-Jeong Won, 2017 [43]	Korea	LTBI	Patients with LTBI, individuals without infection, and cases of TB from a university hospital	15	PTB = 48 NI = 13	Median (range) LTBI: 52.0 (36-75) NI: 28.9 (16-74) TB QFT+: 73.0 (15-86) TB QFT-: 73.5 (25-89)	LTBI: 46.7 TB: 58.3 NI: 53.8	Not reported	Culture	QuantiFERON-TB Gold In-Tube Test	EGF, eotaxin, G-CSF, GM-CSF, IFN-*α*2, IFN-*γ*, IL-1*α*, IL-1*β*, IL-1RA, IL-2, IL-3, IL-4, IL-5, IL-6, IL-7, IL-8, IL-10, IL-12p40, IL-12p70, IL-13, IL-15, IL-17, IP-10, MCP-1, MIP-1*α*, MIP-1*β*, TNF-*α*, TNF-*β*, VEGF	Unstimulated TB vs. LTBI: TNF-*α* and VEGF. TB vs. LTBI and NI: IL-8, IL-13, INF-*γ*, IL-2, IP-10, and VEGF. Stimulated LTBI and TB vs. NI: GM-CSF, IFN-*γ*, IL-1RA, IL-2, IL-3, IL-13, IP-10, and MIP-1*β*. LTBI vs. TB: EGF, GM-CSF, IL-5, IL-10, and VEGF	Plasma samples from whole blood (unstimulated, antigen-stimulated, or mitogen-stimulated)	ESAT-6, CFP-10, and TB7.7 Time: 16-24 hours	Microbead-based method, Milliplex MAP Human Cytokine/Chemokine 29-plex kits (Millipore, Billerica, CA)

Ditthawat Nonghanphithak, 2017 [20]	Thailand	LTBI	All individuals were from Srinagarind Hospital, Khon Kaen. Healthy persons with a history of TB contact and healthy individuals with no known TB exposure	38	PTB: 48 Early clearance: 162 NI: 39	Mean ± SD LTBI: 45 ± 12 TBA: 52 ± 15 EC: 37 ± 16 HC: 40 ± 14	TB: 35.4 LTBI: 81.6 EC: 66 HC: 82.1	Not reported	Smear (ZN), culture, or a molecular test (Xpert MTB/RIF, clinical diagnosis)	QuantiFERON-TB Gold In-Tube Test	CCL2, CXCL10, IFN-*γ*	Unstimulated NI vs. TB and LTBI: CCL2 TB vs. NI, EC and LTBI: CXCL10 LTBI vs. HC and EC: CXCL10 Stimulated LTBI vs. TB: INF- *γ*. TB vs. EC and HC: INF- *γ*, CXCL10. TB and LTBI vs. NI: CXCL10	Nonstimulated and antigen-stimulated PBMC culture supernatants	ESAT-6, CFP-10, and TB7.7 Time: 24 hours	ELISA, BioLegend (California, USA)

Marco Pio La Manna, 2018 [55]	Italy	LTBI	Patients with active TB, health workers, and people with LTBI in a hospital	32	PTB: 27 NI: 20 Others non-TB pulmonary infections: 20	Range LTBI: 17-84 TB: 17-82 Non-TB: 24-76 NI: 21-68	LTBI: 25 TB: 22 Non-TB: 40 NI: 30	Not reported	Culture or GeneXpert MTB/RIF from biopsy specimens and/or biological fluids	QuantiFERON-TB Gold In-Tube Test, TST	IL-1*α*, IL-1*β*, IL-1ra, IL-2, IL-2Ra, IL-3, IL-4, IL-5, IL-6, IL-7, IL-8, IL-9, IL-10, IL-12(p40), IL-12(p70), IL-13, IL-15, IL-16, IL-17, IL-18, IFN-*α*2, IFN-*γ*, TNF-*α*, TNF-*β*, TRAIL, CXCL1 (GRO-*α*), CXCL9 (MIG), CXCL10 (IP-10), CXCL12 (SDF-1*α*), CCL2 (MCP-1), CCL3 (MIP-1*α*), CCL4 (MIP-1*β*), CCL5 (RANTES), CCL7 (MCP-3), CCL11 (eotaxin), CCL27 (CTACK), G-CSF, M-CSF, GM-CSF, SCF, SCGF-*β*, LIF, MIF, FGF-*β*, b-NGF, PDGF-BB, VEGF, HGF	Unstimulated LTBI vs. NI and non-TB: IL-1*β*, IL12p70, and VEGF TB vs. NI and non-TB: PDGF-BB, IL-1*β*, IL-2, IL-8, IL12p70, MCP-1, and LIF. Stimulated TB/LTBI vs. non-TB: IL12-p40, IL-2ra, SCF, TRAIL, IL-2, IFN-*γ*, IP-10, b-NGF, LIF, and MIG. TB vs. non-TB: IFN*α*2, IL-3, and TNF-*β*. LTBI vs. non-TB: IL-13. LTBI and non-TB vs. TB: MIF	Plasma samples from whole blood (unstimulated, antigen-stimulated, or mitogen-stimulated)	ESAT-6/CFP-10 Time: 16–24 hours	Microbead-based method; there is no information

Leonar Arroyo, 2018 [44]	Colombia	LTBI	TB case contacts and TB cases	20	PTB: 21	Median (IQR) LTBI: 38.5 (26.75-52.75) TB: 28 (24-41)	LTBI: 45 TB: not reported	Not reported	Smear (ZN)	Positive response (≥22 pg/ml) to the CFP10 antigen of Mtb and the absence of clinical symptoms compatible with clinical TB	IFN-*γ*	Stimulated LTBI vs. TB: IFN-*γ* in response to all antigens	Nonstimulated and antigen-stimulated PBMC culture supernatants	Mtb DosR (Rv1737c, Rv2029c, and Rv2628) and Rpf (Rv0867c and Rv2389c) antigens Time: 7 days	Microbead-based method, Millipore (Millipore, Billerica, MA, USA)

EPTB: extrapulmonary TB; PTB: pulmonary TB; TB/NRCF: the article does not report the clinical form of TB. ^∗^An example of paper 1 for the row increased immune parameter interpretation: unstimulated, TB vs. NI, and LTBI: IL-10 and IL-6 mean that in an unstimulated sample, IL-10 and IL-6 were increased in TB compared to not infected individuals and persons with LTBI.

**Table 2:** Substances evaluated for ability to differentiate people with active and latent tuberculosis and uninfected with TB.

Interleukins	IL-1, IL-1*α*, IL-1*β*, IL-2, IL-3, IL-4, IL-5, IL-6, IL-7, IL-8, IL-9, IL-10, IL-12, IL-12 (p40/70), IL-12 (p40), IL-12 (p70), IL-13, IL-15, IL-17, IL-17A, IL-17E, IL-17F, IL-18, IL-21, IL-22, IL-23, IL-25, IL-27, IL-28A, IL-31, IL-33
Growth factors	PDGF, PDGF-BB, EGF, PGF2*α*, FGF, TGF-*α*, TGF-*β*1, G-CSF, CSF2, CSF3, GM-CSF, VEGF, VEGF-A, SFC, *β*-NGF, basic FGF
Interferon	INF-*γ*, IFN-*α*, IFN-*α*2
Receptors	IL-1RA, IL-2R, sIL-2R*α*, TNF-*α*R, IL-1R1, IL-1R2
Tumor necrosis factors	TNF-*α*, TNF-*β*, TNF-SF10 (TRAIL)
Alpha chemokines	CXCL5 (ENA-78), CXCL6 (GCP-2/LIX), CXCL8 (IL-8), CXCL9 (MIG), CXCL10 (IP-10), CXCL11 (I-TAC), CXCL12 (SDF-1*α*+*β*), CXCL13 (BCA-1)
Beta chemokines	CCL1 (I-309), CCL2 (MCP-1), CCL3 (MIP-1*α*), CCL4 (MIP-1*β*), CCL5 (RANTES), CCL7 (MCP-3), CCL8 (MCP-2), CCL11 (eotaxin), CCL13 (MCP-4), CCL15 (MIP-1*δ*), CCL17 (TARC), CCL20 (MIP-3*α*), CCL21 (6Ckine), CCL24 (eotaxin-2), CCL26 (eotaxin-3), CCL27 (CTACK)
Delta chemokines	CX3CL1 (fractalkine)
Others	ICAM-1 (CD54), sCD163, sCD14, sCD40L, I-TAG, MIF, LIF, LXA4, 15-Epi-LXA4

## Data Availability

All data generated or analyzed during this study are included in this published article [and its Additional files 1, 2, 3, 4 5 and 6].

## Abbreviations

TB: Tuberculosis
LTBI: Latent tuberculosis infection
EPTB: Extrapulmonary TB
PTB: Pulmonary TB
NI: No
TST: Tuberculin skin test
HIV: Human immunodeficiency virus
NIH: National Institutes of Health
IGRAs: Interferon-gamma release assays
INF: Interferon
IL: Interleukins
TNF: Tumor necrosis factor
PRISMA: Preferred Reporting Items for Systematic reviews and Meta-Analyses
ESAT-6: Early secretory antigenic target-6, Rv3875
CFP-10: Culture filtrate protein-10, Rv3874
PPD: Purified protein derivatives
IP: Immune parameter.

## Supplementary Materials

Supplementary Materials Additional file 1: search strategies conducted in the systematic review. Additional file 2: summary of excluded studies and the reasons. Additional file 3: quality of cohort studies (risk of bias evaluation). Additional file 4: quality of cross-sectional studies (risk of bias evaluation). Additional file 5: immune factors, production cell, diana cell, and effect in different tuberculosis stages. Additional file 6: concentration of immune factors in persons with different tuberculosis stages in the papers included in the systematic review.
